# Thyroid cancer harboring *PTEN* and *TP53* mutations: A peculiar molecular and clinical case report

**DOI:** 10.3389/fonc.2022.949098

**Published:** 2022-09-02

**Authors:** Carla Colombo, Gabriele Pogliaghi, Delfina Tosi, Marina Muzza, Gaetano Bulfamante, Luca Persani, Laura Fugazzola, Valentina Cirello

**Affiliations:** ^1^ Division of Endocrine and Metabolic Diseases, Istituto Auxologico Italiano Istituto di Ricovero e Cura a Carattere Scientifico (IRCCS), Milan, Italy; ^2^ Department of Pathophysiology and Transplantation, University of Milan, Milan, Italy; ^3^ Laboratory of Endocrine and Metabolic Research, Istituto Auxologico Italiano IRCCS, Milan, Italy; ^4^ Unit of Human Pathology, Department of Health Sciences Santi Paolo e Carlo Medical School, University of Milan, Milan, Italy; ^5^ Department of Biotechnology and Translational Medicine, University of Milan, Milan, Italy

**Keywords:** aggressive follicular thyroid cancer, *PTEN*, *TP53*, mismatch repair proteins, microsatellite instability, tyrosine kinase inhibitor, Sorafenib

## Abstract

To date, the molecular mechanisms that underline aggressiveness and resistance to tyrosine kinase inhibitors in some thyroid carcinomas (TCs) are not known yet. We report the case of a young patient with a metastatic poorly differentiated (PDTC) and follicular thyroid carcinoma (FTC) refractory to conventional therapies and to Sorafenib. The patient, despite an initial partial response, died of progressive disease 21 months after diagnosis. The genetic analysis performed on the primary tumor and on lymph nodes and distant metastases allowed to identify a frameshift mutation (p.P248Tfs*5) in the *PTEN* gene, never described in TC. This mutation was present in the primary tumor and, with a lower allelic frequency, in metastases diagnosed after treatment with Sorafenib. Mutations in *TP53* (p.C135Y and c.920-2A>G previously detected in anaplastic carcinomas and p.M133R never found in TC) were also detected in the primary tissue together with a mono-allelic expression of the p.C135Y mutant at RNA level. At metastatic sites level, we found only the *TP53* splicing mutation c.920-2A>G. The presence of defects in mismatch repair (MMR) proteins and genomic instability was also evaluated. The primary tumor showed a partial expression of MMR proteins together with a strong genomic instability. In conclusion, we demonstrated that the rare combination of somatic *PTEN* and *TP53* mutations in a patient with a metastatic FTC, together with the presence of tumor heterogeneity and genomic instability, might be associated with a high tumor aggressiveness and resistance to treatments.

## Introduction

Well-differentiated thyroid carcinomas (WDTCs) are efficiently treated by surgery and radioiodine. On the contrary, poorly differentiated thyroid cancer (PDTC) and anaplastic thyroid cancer (ATC) are refractory to radioiodine therapy, and, in recent decades, tyrosine kinase inhibitors (TKIs) with angiogenetic and molecular targets were developed and used for these cases (1). The molecular mechanisms that generate thyroid cancer (TC) dedifferentiation are still unclear. Recently, next-generation sequencing (NGS) studies unraveled PDTCs and ATCs mutational landscapes, supporting the model of multistep tumorigenesis whereby PDTCs and ATCs arise from WDTCs through stepwise accumulation of additional genetic abnormalities, with prognostic and possible therapeutic relevance (2, 3). To date, one of the best characterized genetic alterations leading to the development of poorly and undifferentiated thyroid cancers is the loss of p53 tumor suppressor. The *TP53* gene codifies for a master regulatory protein, also known as “guardian of genome”, involved in different cellular processes such as apoptosis, DNA repair, cell cycle arrest, and cellular senescence (4). The p53 protein has a key role in the maintenance of genetic stability and, thus, in preventing tumor development. *TP53* mutations, usually located in the region between exons 5 and 8, have been described in about 50% of human cancers. Whereas WDTC are rarely (<10%) *TP53* mutated, more than 70% of PDTC/ATCs are associated with *TP53* mutations (5). Almost all p53 mutations impaired p53 transcriptional activity and are not only important for tumor progression but also in the response to chemotherapy, to radioiodine therapy, and to TKIs treatment (5, 6). Interestingly, two mismatch repair genes, *MLH1* and *PMS2*, have been identified as targets for p53 in normal fibroblasts (7). The mismatch repair (MMR) system recognizes mismatched bases in double-stranded DNA and initiates the repair process. The identification of MLH1 and PMS2 as direct targets for p53 defines a signaling pathway that couples two important cellular guardian pathways, growth arrest, and apoptosis (7). Another tumor suppressor gene often mutated in human cancers, and also in aggressive TCs, is *PTEN* (phosphatase and tensin homolog). PTEN, through its lipid phosphatase activity inhibiting the PI3K/AKT pathway, regulates many cellular processes, including proliferation, survival, energy metabolism, cellular architecture, and motility (8). Mutations result in a non-functional or absent PTEN protein and are relatively common in ATCs, followed by PDTCs, and uncommon in follicular thyroid cancer (FTC) (9). Although TKIs are now available for aggressive TCs treatment (1, 10), additional strategies are currently being investigated, using the ability to modulate epigenetic changes in cancer DNA, restore the transcriptional activity of mutant p53, and block signal transduction downstream of different p53 family members (11). Immune checkpoint inhibitors, already used in colorectal cancer patients with high level of microsatellite instability or with defects in one of the MMR genes (12), might be also explored in the future. In this study, we reported the peculiar clinical and molecular characterization of a 35-year-old male patient who died of a metastatic FTC refractory to surgery and radioiodine and Sorafenib treatment and harboring *PTEN* and *TP53* mutations in a context of tumor heterogeneity and genomic instability.

## Case description

**Figure 1:**
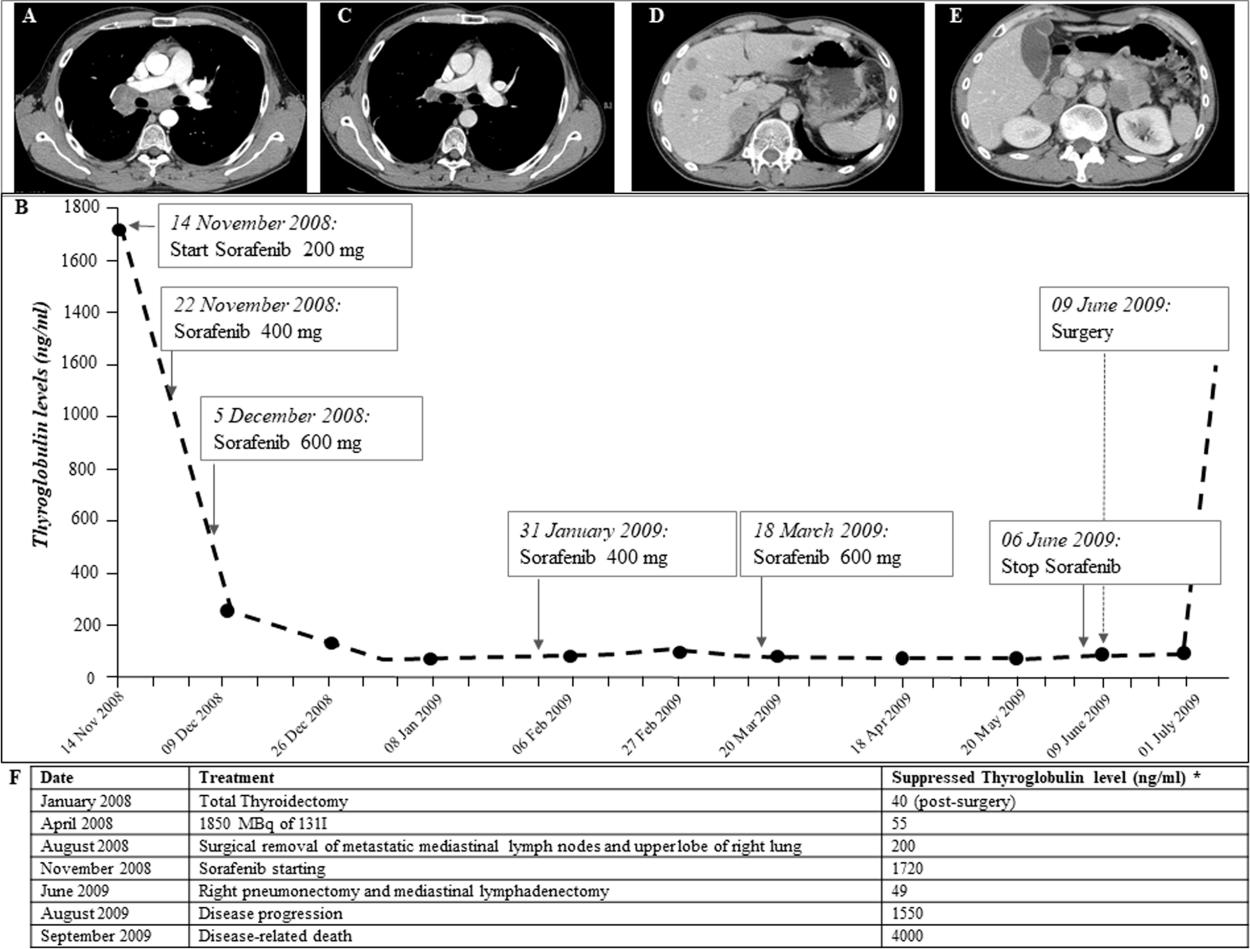
**(A)** CT scan performed on November 2008, before Sorafenib is started. Large lymph node metastases (between 2 and 5.5 cm in diameter) localized in the right hilar region across the pulmonary artery and the main bronchus. **(B)** Thyroglobulin (Tg) biomarker trend during Sorafenib treatment and after surgical removal of metastases performed after Sorafenib treatment (anti-Tg antibodies persistently negative): Tg values were significantly reduced in the first few weeks of Sorafenib therapy and remained low throughout treatment on different doses. In July 2009, after surgical removal of lymph node and lung metastases, Tg values rose suddenly. **(C, D)** CT scan performed on July 2009, after following Sorafenib withdrawal and surgical removal of lymph node and lung metastases. Ten liver metastases of 1–2.5 cm size and adrenal metastases (5 cm on the left and 4 cm on the right) were observed. **(E)** CT scan performed on January 2009, during Sorafenib treatment, showing significant reduction in lymph node metastases (maximum diameter 1 cm). **(F)** Chronological description of serum suppressed thyroglobulin levels and treatments carried out for thyroid carcinoma.

In January 2008, a 35-year-old man without a family history of benign/malignant thyroid diseases or other tumors had noticed the appearance of a rapidly growing nodule in the neck. The patient had never had any relevant diseases or tumors, thus excluding the DICER1 syndrome, and had therefore never undergone external radiotherapy at the neck level. An ultrasound of the neck showed a hypoechogenic thyroid nodule of about 35 mm in the right lobe. Therefore, the patient underwent a thyroid needle aspiration for cytological examination. The patient was submitted, for a suspected cytological result, to total thyroidectomy. According to the WHO 2022 classification, histological examination showed a 35-mm right lobe extensively invasive, necrotic, and angioinvasive PDTC (80%) with widely invasive FTC areas with pleomorphic nuclei (20%) (pT3NX, according to the 7th TNM edition) (13, 14). The tissue analyzed shows Turin criteria (14): solid/trabecular/insular growth pattern, no nuclear cytology features of PTC, presence of tumor necrosis, mitotic count of 8/2 mm^2^, convoluted nuclei, and absence of anaplastic features. In addition, Ki-67 immunostaining showed a proliferation index >5% in both the primary tumor and the distant metastasis (**Supplementary Figure 1**). Moreover, at diagnosis, a CT scan showed the presence of a small lung suspicious nodule and multiple metastatic lymph nodes (laterocervical, paratracheal, and mediastinal) and particularly a 5-cm lymph node metastasis located in the right hilar region across the main bronchus. In April 2008, 1,850 MBq of 131I was administered, and the total body scan showed two small thyroid residues and laterocervical adenopathy, showing instead a radioiodine refractoriness of mediastinal and lung metastases. In August 2008, 3 months after initial treatments, the patient was submitted to a thoracic surgery in order to remove progressive lung metastases and several enlarging mediastinal lymph nodes, conditioning dyspnea, chest pain, dysphagia, and fatigue. However, the metastases were unresectable, only a few lymph nodes were debulked, and the upper lobe of the right lung was removed. Histological examination showed massive lymph node and lung metastases of PDTC. Thus, in November 2008, due to the presence of progressive, symptomatic, iodine-refractory, unresectable distant metastases (**Figure 1A**), after informed consent of the patient, we started treatment with Sorafenib, the only TKI available at the time (1, 15). Due to major drug-related side effects that developed (diarrhea, pruritus, fatigue, weight loss, hand–foot syndrome, musculoskeletal pain, and tachycardia), the maximum dosage reached by the patient was 600 mg/day, which was maintained without any withdrawal during 7 weeks. During Sorafenib treatment, lymph node and lung metastases showed a significant volume reduction, and serum thyroglobulin (Tg) decreased from 1,700 to 55 ng/ml (**Figure 1B**). The reduction of Sorafenib to 400 mg daily was associated with an immediate increase in Tg values, without a substantial reduction in side effects. The dose was then increased to 600 mg/day with a new decrease in Tg levels (**Figure 1B**). In June 2009, given the significant reduction of metastases burden (**Figure 1C**), the patient underwent right pneumonectomy and mediastinal lymphadenectomy. The histological examination showed lymph node and lung PDTC metastases. In July 2009, after surgery, serum Tg levels increased progressively, and a total body CT scan, negative for neck and chest metastases, showed multiple liver and adrenal metastases (**Figures 1D–F**). The patient’s general conditions deteriorated rapidly, so no further treatments were possible, and he died in September 2009.

## Molecular and Protein expression Analyses

**Figure 2:**
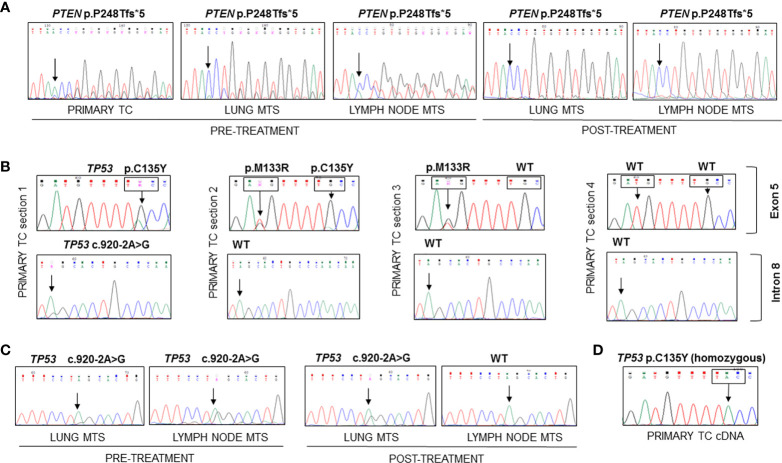
Sequencing of the PCR amplicons corresponding to exon 7 of PTEN and to exons 5 and 9 of TP53 genes and of RT-PCR amplicon corresponding to exon 5 of TP53 in FFPE and frozen samples. **(A)** The PTEN p.P248Tfs*5 mutation was found in heterozygosity in the DNA extracted from FFPE primary TC and lung and lymph node metastases samples obtained before and after Sorafenib treatment. **(B)** Different mutational patterns observed for TP53 in four sections of primary TC. The first sample showed the presence of TP53 p.C135Y mutation in exon 5 and c.920-2A>G splicing variant in intron 8, the second harbored two TP53 mutations (p.M133R and p.C135Y), the third had only the p.M133R mutant, while the last had no mutations. **(C)** The c.920-2A>G splicing variant was found in all metastases analyzed with the exception of the lymph node metastasis obtained after Sorafenib treatment. **(D)** The analysis of TP53 transcript encompassing exon 5 showed the presence of the C135Y mutation in homozygous state in the cDNA obtained from frozen primary TC. TC, thyroid cancer; MTS, metastasis.

At the time of diagnosis, the only molecular analyses available in our laboratory concerned the search for *BRAF* and *RAS* mutations and *ret/PTC* rearrangements, which were negative in this patient. Therefore, now that the molecular analysis in our laboratory has been extended to numerous genes involved in thyroid carcinogenesis by means of both mass spectrometry and Sanger sequencing, we have decided to re-analyze this particular case. The molecular analysis of the DNA obtained from the formalin-fixed paraffin-embedded (FFPE) primary TC revealed the presence of a frameshift variant in exon 7 of *PTEN* (c.741dupA, p.P248Tfs*5) (**Figure 2A**) and a missense mutation in exon 5 of *TP53* gene (c.404G>A, p.C135Y) in heterozygosis, both absent in the corresponding contralateral normal thyroid tissue (*data not shown*). Analyzing different frozen primary TC sections, we confirmed the presence of the *PTEN* p.P248Tfs*5 mutation in all specimens (*data not shown*). On the other hand, a heterogeneous *TP53* molecular profile was observed in these samples: one harbored the *TP53* p.C135Y mutant together with a splicing mutation in intron 8 (c.920-2A>G), the second harbored the *TP53* p.C135Y variant together with another missense variant (c.398T>G, p.M133R), the third sample had only the p.M133R mutation, and the last had no *TP53* mutations (**Figure 2B**). Evaluating the presence of these variants in patient’s FFPE lung and lymph node metastatic samples, the *PTEN* frameshift was detected in all tissues obtained pre- and post-Sorafenib treatment but, in the latter, at very low allelic frequency (**Figure 2A**). On the contrary, the two *TP53* missense mutations in exon 5 were absent in all metastatic samples analyzed, while the splicing variant c.920-2A>G was present in lung metastases obtained both pre- and post-treatment and in the lymph node metastasis before starting Sorafenib (**Figure 2C**). Interestingly, at RNA level the *TP53* p.C135Y mutation was found in homozygosis in the primary TC tissue (**Figure 2D**). The DNA recovered from the sample harbored the p.C135Y mutation in heterozygosis.

**Figure 3:**
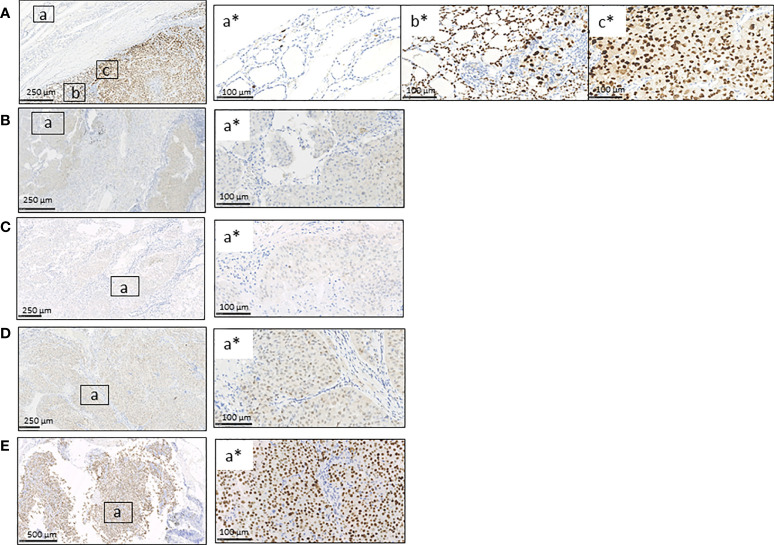
Immunohistochemistry for p53 protein in FFPE primary TC, contralateral normal thyroid tissue, and lung and lymph node metastases obtained before and after Sorafenib treatment. **(A)** The immunostaining for p53 was wild type in the contralateral normal thyroid tissue (inset a*) and abnormal/over-expressed with high nuclear expression in primary TC, both FTC/PDCT (inset b*) and PDTC areas (inset c*). **(B–D)** Lung metastases obtained before **(B)** and after Sorafenib treatment **(D)** and lymph node metastasis before TKI **(C)** showed an abnormal/cytoplasmatic p53 staining with low nuclear expression (for each is shown a selected inset area). **(E)** The lymph node metastasis obtained after the TKI treatment showed an abnormal/over-expressed p53 pattern with high nuclear expression. FTC, follicular thyroid cancer; PDTC, poorly differentiated thyroid cancer; NT, normal thyroid; TKI, tyrosine kinase inhibitor. Scale bar are shown for each images.

We then investigated the expression of p53 at protein level by immunohistochemistry. A peculiar pattern was observed in tissues analyzed: p53 was abnormal/over-expressed with high nuclear expression in primary TC (both FTC and PDTC areas) and normal in the contralateral normal thyroid tissue (**Figure 3A**). As far as metastases are concerned, all metastatic tissues had an abnormal/cytoplasmatic p53 staining with low nuclear expression (**Figures 3B–D**) with the exception of the lymph node obtained after Sorafenib treatment showing an abnormal/over-expressed p53 pattern with high nuclear expression (**Figure 3E**).

**Figure 4:**
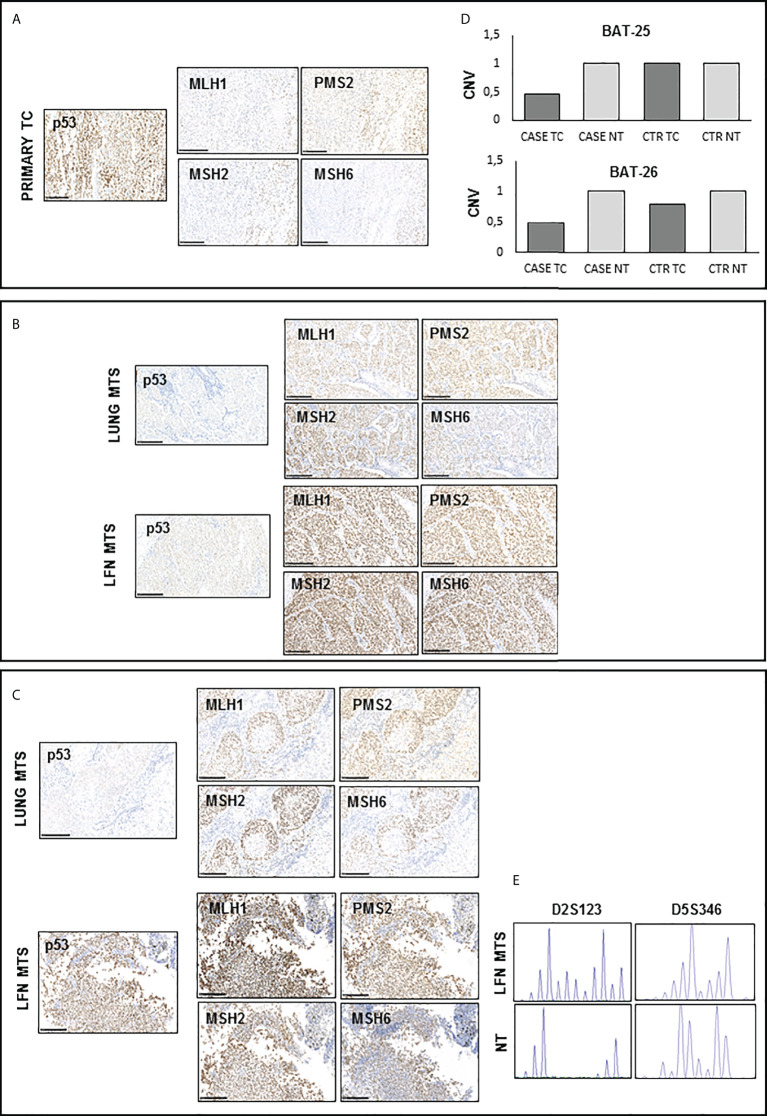
Immunohistochemistry for p53 and DNA mismatch repair (MMR) proteins and microsatellite instability (MSI) detection in available samples before and after Sorafenib treatment **(A, C, D)** A positive nuclear staining was observed for MHL1, MSH2, MSH6, and PMS2 proteins in all analyzed tissues samples. However, primary TC shows light staining and subclonal loss of expression in all MMR proteins. **(B)** CNV analysis shows the presence of a LOH for both BAT-25 and BAT-26 loci in the primary TC of our patient. **(E)** Peak analysis of D2S123 and D5S346 loci, performed using Genemapper 5 software, is clearly differently shaped in the lymph node obtained after Sorafenib treatment with respect to those of and contralateral normal thyroid tissue DNA, indicating a high microsatellite instability. TC, thyroid cancer; MTS, metastasis; NT, normal thyroid; CNV, copy number variation.

Finally, evaluating the expression of mismatch repair proteins (MMRs), a positive nuclear staining was observed for all these markers (MHL1, MSH2, MSH6, and PMS2) in the tissues analyzed (**Figures 4A–C**), although not all nuclei in the primary tumor showed the expression of MMR antigens (**Figure 4A**). It is interesting to note that there is an almost total loss of MMR proteins in the tumor area corresponding to PDTC (**Supplementary Figure S2**). The immunostaining score for each protein analyzed is reported in **Supplementary Table S1**. Interestingly, the analysis of microsatellite instability (MSI) status showed no amplification of BAT25 and BAT26 loci in the primary TC (**Supplementary Table S2**). Copy number variation (CNV) analysis showed the presence of a loss of heterozygosity (LOH) for both loci in the primary TC (**Figure 4D**). On the other hand, a high MSI for D2S123 and D5S346 loci was found only in the lymph node metastasis obtained after Sorafenib treatment, as shown in **Figure 4E**.

## Discussion

To date, the molecular mechanisms associated with TC aggressiveness and resistance to TKIs treatments are not well understood. In the present study, we reported the case of a 35-year-old male patient with metastatic poorly differentiated (PDTC) and follicular thyroid carcinoma (FTC) treated with surgery, radioiodine, and Sorafenib. The patient, despite an initial partial response, died of progressive disease 21 months after diagnosis.

Molecular analyses of primary tumor and metastatic tissues showed the co-occurrence of a *PTEN* frameshift variant (p.P248Tfs*5) together with three *TP53* mutations (p.C135Y, p.M133R, and c.920-2A>G) in some, but not all, samples analyzed. We are tempted to speculate that TP53 mutations occur in poorly differentiated tumor area, since 80% of the primary TC is composed by PDTC. The co-occurrence of both *PTEN* and *TP53* mutations in thyroid and other cancers has already been reported (16, 17). In particular, the *PTEN* p.P248Tfs*5 variant was previously identified at somatic level in several solid tumors (COSM4986), but never in sporadic TC. On the other hand, the *TP53* p.C135Y variant and the splicing mutation c.920-2A>G were already described in ATC [3, 18] and in other solid and hematopoietic tumors (COSM10801 and COSM33650). Finally, the *TP53* p.M133R variant was reported at germline level in Li–Fraumeni syndrome and at somatic level in solid and hematopoietic tumors (COSM43730), but never in TC. It is possible to speculate that TP53 mutations.

Interestingly, the *PTEN* mutation is constantly found in primary TC, and all metastases analyzed were consistent with its clonal origin. It is worth to note that the allelic frequency of this mutation is lower in metastases obtained after Sorafenib treatment with respect to other samples, indicating a potential effect of the TKI treatment on tumor clones harboring the *PTEN* frameshift. On the other hand, a variable mutational pattern for *TP53* was observed in primary TC and metastases samples (sections with either one or two or three mutations or entirely wild type for TP53). We excluded that these mutations are passenger ones, as they were previously reported in TC and other cancers and known to be non-functional pathogenic variants (p.C135Y and p.M133R, https://tp53.isb-cgc.org) or predicted to be likely pathogenic/pathogenic (c.920-2A>G, https://www.ncbi.nlm.nih.gov/clinvar). The finding of three mutations affecting the *TP53* gene is not surprising, since FTC exhibits remarkable genomic instability evidenced by *TP53* hyper-mutability (5). *TP53* mutations seem to be subclonal, each present in only a subset of malignant cells, contributing to heterogeneity within the tumor and potentially to treatment resistance (19, 20). Indeed, preclinical and clinical evidence suggests that cancer harboring *TP53* mutations are often resistant to TKI inhibitors (6, 21–25). Intriguingly, the p.C135Y mutation known to be a non-functional, dominant negative hot spot mutant (https://tp53.isb-cgc.org) (26) was found in homozygosis at RNA level in primary TC, suggesting a monoallelic expression (MAE) of the mutated allele. LOH of cancer-associated genes at DNA level is a common and important mechanism in carcinogenesis, but MAE at RNA level is a much less understood phenomenon. MAE may precede or enhance a mutation by expression of only the mutant or disease-related allele, having a role in tumor progression and clinical implications. High rate of MAE was previously observed in progressive brain tumors harboring *TP53* mutations (27, 28), but never in TC. The finding of the *TP53* p.M133R mutation in our patient is also intriguing, since germline mutations affecting the codon 133 cause the loss of the Δ133p53 isoforms and are frequently implicated in the development of Li–Fraumeni and Li–Fraumeni-like cancer predisposing syndromes (29). Although the precise functions of these isoforms is still poorly understood, syndromic forms of breast cancer are strongly associated with the loss of codon 133, indicating that the expression of Δ133p53 isoforms is critical for regulating p53 activity and carcinogenesis in some tissues (30). The presence of these two *TP53* missense mutations within exon 5 in primary TC is in agreement with the nuclear p53 protein expression pattern. On the other hand, the *TP53* c.920-2A>G splicing mutant is likely unable to enter the nucleus and accumulate in the cytoplasm, and, indeed, an abnormal/cytoplasmatic p53 staining was observed in all tissues harboring this mutation. The lymph node metastasis obtained post-Sorafenib is the only tissue that, despite an abnormal/over-expressed p53 staining with high nuclear expression, does not harbor *TP53* mutations. For this sample, we cannot exclude the presence of mutations in intronic/regulatory regions or a LOH of the *TP53* gene as far as the presence of alterations in other proteins of the DNA repair pathway that finally cause the inactivation of p53 protein. The possible involvement of defects in the DNA MMR proteins was also suspected, since the patient had multiple mutations. In our study, MMR proteins resulted to be functional in all tissue samples, although the primary TC showed a partial loss of MMR expression as already reported in a FTC case (31). Defects in MMR proteins are responsible for genomic instability, which can be evidenced by alterations in microsatellites markers. The primary TC, in which we detected a partial loss of MMR proteins, showed an LOH for BAT-25 and BAT-26 microsatellites. LOH for these loci were not shared by any metastatic site, but surprisingly, the lymph node metastasis obtained after Sorafenib treatment showed a MSI-High (MSI-H) for other two microsatellites (D2S123 and D5S346), but a normal MMR proteins expression. The discordant pattern of CNV for microsatellites between primary and metastatic sites may be explained by independent clonal evolution selected during the metastatic process. Moreover, the finding of MSI-H and MMR-proficient in the lymph node metastasis after Sorafenib is not surprising. Indeed, it is possible that some missense mutations in MMR genes can lead to functional inactivation of the corresponding protein without affecting its stability, antigenicity and expression level (32) or that some MSI-H tumors derive from alterations of MMR pathway-related proteins are not detectable by current technologies (33). It is exciting to find that the two tumor samples with abnormal nuclear expression of p53 protein (primary tumor and lymph node metastases after Sorafenib) show a high genomic instability, highlighting a strong relationship between MMR and the role of p53 in regulation of the cell-cycle arrest/apoptosis decision processes when DNA damage overwhelms a critical threshold. As recently reported (34, 35), subclonal expansions seem common in thyroid cancer cases with aberrant DNA repair with a selection of highly aggressive clones that will progress as what we observed in our patient.

Unfortunately, the patient’s general conditions deteriorated rapidly after surgery in June 2009, and thus, distant liver and adrenal metastases were not available for further molecular characterization. It is well-known that TKIs exert their effect through a cytostatic action, and, once started, a continuative treatment is needed to maintain a response to the disease. When the TKI treatment is stopped, as it happened for our patient, the escape phenomenon is observed, and the progression of the disease can become even more rapid.

## Conclusion

In conclusion, we demonstrated the presence of genomic heterogeneity and instability in a patient with metastatic poorly differentiated (PDTC) and follicular thyroid carcinoma (FTC) refractory to all treatments. The rare combination of *PTEN* and *TP53* mutations seems to be associated with a particular tumor aggressiveness and maybe with a possible resistance to TKI. This case report highlights the importance of characterizing both primary tumor and metastases at molecular level to predict the response to treatments. Indeed, the tumor heterogeneity can evolve during tumor progression or as a consequence of drug-dependent selection of a pre-existing or newly acquired resistant clones. For this reason, further studies are needed, and new therapeutic strategies will be explored, such as drugs able to restore the transcriptional activity of mutant p53 or immune checkpoint inhibitors useful in cancers with high level of microsatellite instability or with defects in MMR genes.

## Data availability statement

The original contributions presented in the study are included in the article/**Supplementary Material**, further inquiries can be directed to the corresponding author.

## Ethics statement

This study was reviewed and approved by Istituo Auxologico Italiano. The patients/participants provided their written informed consent to participate in this study.

## Author contributions

CC provided clinical details. GP performed the molecular analysis of other genes, the analysis of microsatellites status, and of copy number variation. DS performed immunohistochemistry. MM performed PTC mass array. GB revised immunohistochemical results. VC conceived and designed the study; she performed molecular analysis of TP53 gene and transcript. VC, CC, LP, and LF wrote, edited, and reviewed the manuscript. All authors were involved in analyzing the data, writing the paper, and had final approval of the submitted and published versions.

## Funding

This study was partially funded by Ricerca Corrente Istituto Auxologico Italiano IRCCS (PTC-array, 05C825_2018 and THYCANC, 2022_03_08_03) and by the Italian Ministry of University and Research (PRIN 2017-2017YTWKWH).

## Acknowledgments

The author acknowledge the support of the APC central fund of the university of Milan.

## Conflict of interest

The authors declare that the research was conducted in the absence of any commercial or financial relationships that could be construed as a potential conflict of interest.

## Publisher’s note

All claims expressed in this article are solely those of the authors and do not necessarily represent those of their affiliated organizations, or those of the publisher, the editors and the reviewers. Any product that may be evaluated in this article, or claim that may be made by its manufacturer, is not guaranteed or endorsed by the publisher.

## Supplementary material

Supplementary Figure 1 Hematoxylin and eosin (H&E) and Ki-67 stainings performed on the primary thyroid cancer section showing Turin criteria. H&E staining showed the presence of **(A)** pleomorphic nuclei typical of follicular thyroid carcinoma, **(B)** convoluted nuclei, **(C)** tumor necrosis, **(D)** and a mitotic count of 8/2 mm^2^ (black circle).

Supplementary Figure 2 Representative images of the immunohistochemistry for the DNA mismatch repair proteins MSH6 and MHL1 obtained for the primary thyroid cancer. **(A)** Both MHS6 and MLH1 were expressed in all nuclei of follicular cells in the tumor area corresponding to 20% FTC (**A** and **C**, respectively). On the other hand, the almost total loss of both MSH6 and MLH1 expression was observed in the nuclei of follicular cells in the tumor area corresponding to 80% PDTC (**B** and **D**, respectively).
