# Supplementary figures and images for: Thyroid cancer harboring PTEN and TP53 mutations: A peculiar molecular and clinical case report
Source: Front Oncol. 2022 Sep 2;12:949098. doi: 10.3389/fonc.2022.949098 (PMC9478947; doi:10.3389/fonc.2022.949098)

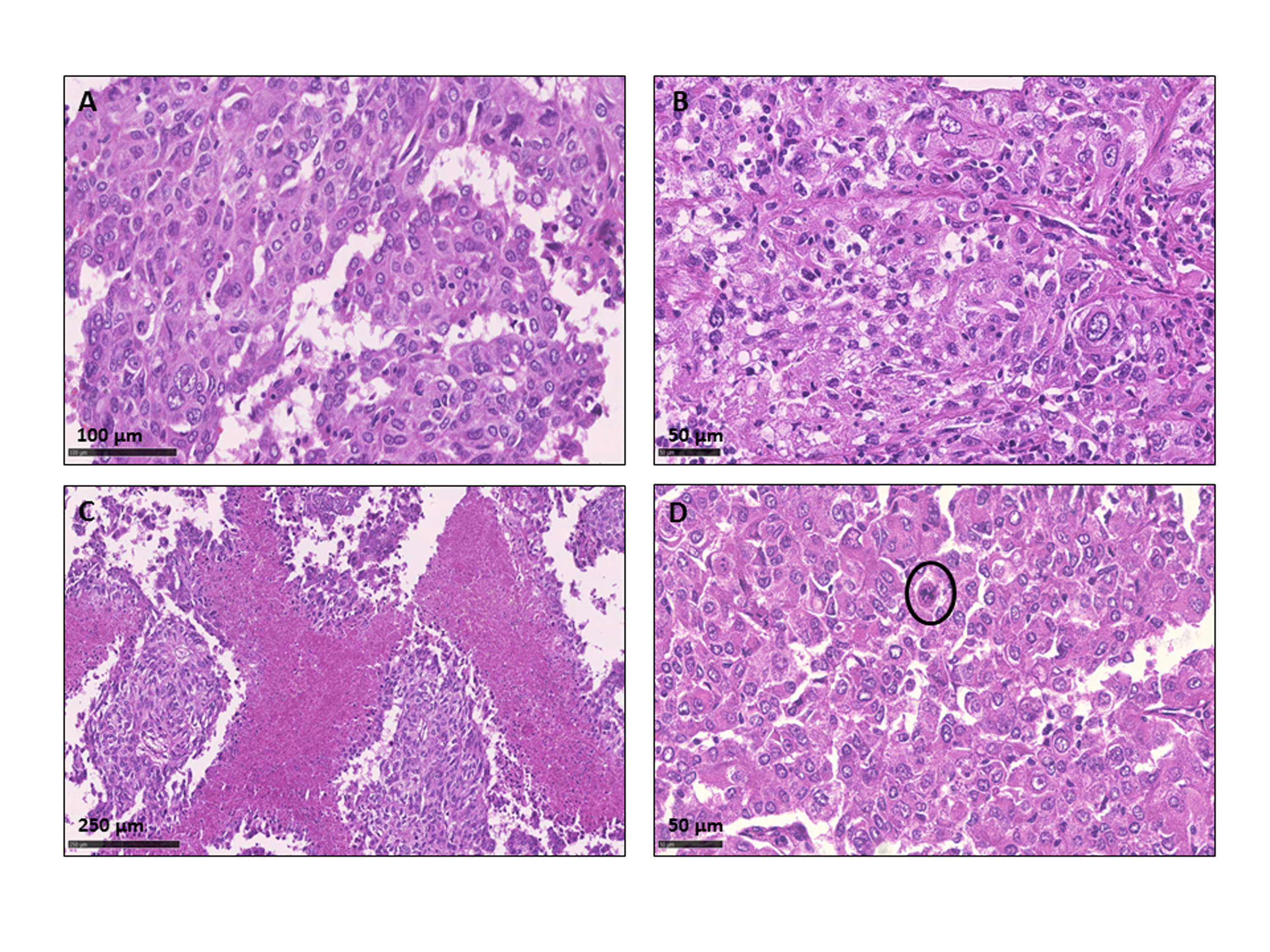

Supplement: Supplementary Figure 1 — Hematoxylin and eosin (H&E) and Ki-67 stainings performed on the primary thyroid cancer section showing Turin criteria. H&E staining showed the presence of (A) pleomorphic nuclei typical of follicular thyroid carcinoma, (B) convoluted nuclei, (C) tumor necrosis, (D) and a mitotic count of 8/2 mm2 (black circle). [file Image_1.tif]

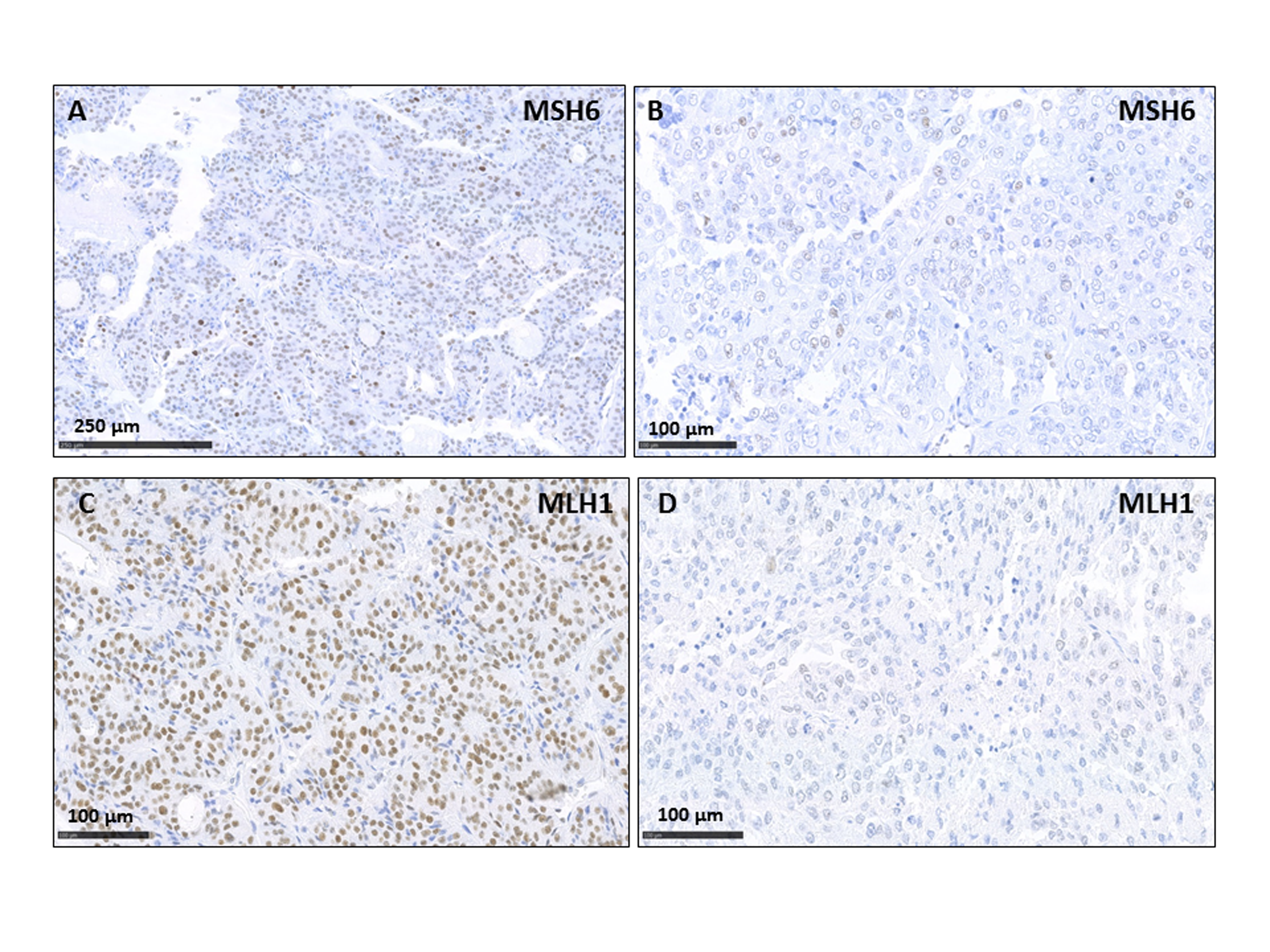

Supplement: Supplementary Figure 2 — Representative images of the immunohistochemistry for the DNA mismatch repair proteins MSH6 and MHL1 obtained for the primary thyroid cancer. (A) Both MHS6 and MLH1 were expressed in all nuclei of follicular cells in the tumor area corresponding to 20% FTC (A and C, respectively). On the other hand, the almost total loss of both MSH6 and MLH1 expression was observed in the nuclei of follicular cells in the tumor area corresponding to 80% PDTC (B and D, respectively). [file Image_2.tif]
