# Supplementary material for: Thyroid cancer harboring PTEN and TP53 mutations: A peculiar molecular and clinical case report
Source: Front Oncol. 2022 Sep 2;12:949098. doi: 10.3389/fonc.2022.949098 (PMC9478947; doi:10.3389/fonc.2022.949098)
Supplement: Supplementary file 3 [file DataSheet_1.docx]

**MATERIAL AND METHODS**

***Nucleic acids extraction***

Genomic DNA (gDNA) was extracted from formalin fixed paraffin embedded (FFPE) tissue samples (primary TC, lung and lymph node metastases) using the RecoverAll Total Nucleic Acid Isolation Kit (Thermo-Fisher Scientific, Waltham, MA, USA) and from frozen tissues using Puregene Core Kit A (Qiagen, Germantown, MD), according to the manufacturer’s protocol. For FFPE samples, hematoxylin and eosin sections were evaluated to define tumor purity (percentage of neoplastic cells), which corresponds to the amount of sample occupied by cancer cells and not by surrounding stromal and immune/inflammatory cells. A minimum of 50% tumor purity was required for sample processing. The tumor purity was 60-70% for primary thyroid cancer (TC); 70-80% and 80% for lung and lymph node metastases before the TKI treatment, respectively; 50% and 60% for lung and lymph node metastases after the TKI, respectively. Total RNA was extracted from available tissues using the Trizol reagent (Thermo-Fisher Scientific) according to manufacturer’s instruction. RNA was quantified by spectrophotometry, and its quality was checked by the analysis of 260/280 nm and 260/230 nm ratios. One microgram of RNA sample was reverse-transcribed using a Superscript reverse transcriptase II Kit (Thermo-Fisher Scientific), with random hexamer mixture as primers.

***PTC Mass-Array assay and molecular analysis of PTEN, TP53 and other genes***

DNA and cDNA were analyzed using the custom PTC-MA assay, as previously described (36, 37). The molecular analysis of genes not included in the PTC-MA assay (*TP53* exons 2-11, *PTEN* exons 5-8, *KRAS* exon 3, *NRAS* exon 1, *PIK3CA* exons 9 and 20, *CTNNB1* exon 3, and *DICER1* exons 24-25) was performed by PCR amplification and direct sequencing, using newly designed primers (sequences on request). *BRAF*K601E mutation was investigated by direct sequencing of exon 15 (38), and *AKAP9/BRAF* and *LMO7/BRAF* fusions were investigated on cDNA, as previously reported (39, 40).

***TP53 transcript analysis***

The analysis of *TP53* transcript corresponding to exon 5 was performed using 100 ng of cDNA and the specific primers encompassing exons 4-5 (F: 5’-TCTGTGACTTGCACGTACTCC-3’) and exons 5-6 (R: 5’-CAGACCATCGCTATCTGAGCA-3’). To evaluate that the quality of the RNAs was high enough, we performed RT-PCR on a housekeeping gene (*ACTB*). The fragment obtained was purified and sequenced.

***Immunohistochemistry analyses of p53 and MMR proteins***

Immunohistochemical studies were carried out on five consecutive sections 3 μm thick tissue using autostainer DAKO Omnis. Tissue sections were incubated for one hour with anti-p53 antibody (DO-7) and with four antibodies against DNA mismatch repair proteins, i.e. anti-MLH1 (ES05), anti-MSH2 (FE11), anti-MSH6 (EP49), and anti-PMS2 (EP51), following manufacturing protocol. All antibodies were purchased from DAKO and were ready to use. Slides were evaluated independently by two experts (D.T., G.B.) and a semiquantitative assessment was scored on a scale from 0 to 3+ (0, staining completely absent; 1+, immunoreactivity in up to 20% of cells; 2+, immunoreactivity in up to 50% and 3+ more than 51% of cells. Image acquisition was performed by NanoZoomer-XR C12000 series (Hamamatsu Photonics®). The magnification of representative slides is reported as a scale bar.

***Microsatellite instability (MSI) detection***

The MSI status was evaluated by PCR amplification of the five Bethesda recommended microsatellites (41). PCR amplification was perfomed using fluorescent FAM-labeled forward primers (Eurofins Genomics Germany GmbH, Ebersberg, Germany), the AmpliTaq Gold 360 kit (Thermo-Fisher Scientific) and about 120 ng of tumor/normal DNA. All reactions included a negative control and two positive controls (DNA from two colorectal cancer samples with high MSI). PCRs were performed using an Applied Biosystems 2720 Thermal Cycler (Applied Biosystems, Foster City, California, USA) following cycling conditions previously reported (42). PCR products were then loaded on a Applied Biosystems 3500 Genetic Analyzer (Life Technologies Holdings Pte Ltd, Singapore) with the addition of 0,5 µl GeneScan 500 ROX Size Standard (Life Technologies LTD, Warrington, UK) and analyzed with GeneMapper® Software 5 (Applied Biosystems). The mobility shift of PCR products from the tumor DNA was compared to that of the corresponding normal thyroid specimen. Samples were considered MSI high (MSI-H) if two or more loci exhibited instability, MSI-low (MSI-L) if only one locus was interested by instability and MS-stable (MSS) if no instability was detected, as previously reported (41).

***Quantitative PCR (qPCR) for the analysis of Copy number variation (CNV)***

The qPCR analysis using SYBR Green reagents (Thermo-Fisher Scientific) was performed to verify the possible loss of heterozygosity (LOH) for BAT-25 and BAT-26 loci in our patient. The CNV analysis was done starting from the DNA extracted from the FFPE primary TC and normal contralateral thyroid samples of our case, and results compared to those of another cancer patient used as internal control. Amplicons and primers for BAT-25 and BAT-26 loci and for PCNT1 control gene were defined using Primer3 (https://primer3.ut.ee/). qPCR analysis was performed in triplicate on the QuantStudio 12K Flex instrument (Applied Biosystems) using the following thermal cycling conditions: 50°C for 2 min and 95°C for 10 min, followed by 40 cycles at 95°C for 15 s and 60°C for 1 min. Results were obtained and processed with the QuantStudio 12K Flex Software (Applied Biosystems). Relative quantification of the amount of DNA was obtained using the 2−ΔΔCt method (43).

**References:**

36. Pesenti C, Muzza M, Colombo C, Proverbio MC, Farè C, Ferrero S, et al. MassARRAY-based simultaneous detection of hotspot somatic mutations and recurrent fusion genes in papillary thyroid carcinoma: the PTC-MA assay. Endocrine (2018) 61:36–41. doi: 10.1007/s12020-017-1483-2.

37. Colombo C, Muzza M, Pogliaghi G, Palazzo S, Vannucchi G, Vicentini L, et al. The thyroid risk score (TRS) for nodules with indeterminate cytology. Endocr Relat Cancer (2021) 28:225–35. doi: 10.1530/ERC-20-051122

38. Fugazzola L, Mannavola D, Cirello V, Vannucchi G, Muzza M, Vicentini L, et al. BRAF mutations in an Italian cohort of thyroid cancers. Clincal Endocrinol (Oxf) (2004) 61:239–43. doi: 10.1111/j.1365-2265.2004.02089.x

39. Ciampi R, Nikiforov YE. Alterations of the BRAF gene in thyroid tumors. Endocrine Pathol (2005) 16:163–72. doi: 10.1385/ep:16:3:163 40. He H, Li W, Yan P, Bundschuh R, Killian JA, Labanowska J, et al. Identification of a recurrent LMO7-BRAF fusion in papillary thyroid carcinoma. Thyroid (2018) 28:748–54. doi: 10.1089/thy.2017.0258

41. Boland RC, Thibodeau SN, Hamilton SR, Sidransky D, Eshleman JR, Burt RW, et al. A national cancer institute workshop on microsatellite instability for cancer detection and familial predisposition: Development of international criteria for the determination of microsatellite instability in colorectal cancer. Cancer Res (1998) 58:5248–57.

42. Berg KD, Glaser CL, Thompson RE, Hamilton SR, Griffin CA, Eshleman JR. Detection of microsatellite instability by fluorescence multiplex polymerase chain reaction. J Mol Diagn (2000) 2:20–8. doi: 10.1016/S1525-1578(10)60611-3

43. Livak KJ, Schmittgen TD. Analysis of relative gene expression data using real-time quantitative PCR and the 2(–delta delta C(T)) method. Methods (2001) 25:402–8. doi: 10.1006/meth.2001.1262
