# Supplementary material for: Thyroid cancer harboring PTEN and TP53 mutations: A peculiar molecular and clinical case report
Source: Front Oncol. 2022 Sep 2;12:949098. doi: 10.3389/fonc.2022.949098 (PMC9478947; doi:10.3389/fonc.2022.949098)
Supplement: Supplementary file 4 [file Table_1.docx]

| **Samples** | **p53** | | | | **Microsatellite instability** | | | |
| --- | --- | --- | --- | --- | --- | --- | --- | --- |
|  | **Low**  **nuclear expression** | **High**  **nuclear expression** | **Cytoplasmatic expression** | **%** | **MLH1** | **MSH2** | **MSH6** | **PMS2** |
| Normal thyroid |  | + |  | 1 | 2 | 2 | 2 | 3 |
| Primary TC |  | + | + | 3 | 2 | 2 | 2 | 3 |
| Lung mts before TKI | + |  | + | 1 | 3 | 3 | 3 | 3 |
| Lymph node mts before TKI | + |  | + | 3 | 3 | 3 | 3 | 3 |
| Lung mts after TKI | + |  | + | 2 | 3 | 3 | 3 | 3 |
| Lymph node mts after TKI |  | + | + | 3 | 3 | 3 | 3 | 3 |

**Supplementary Table 1:** Semiquantitative assessment of staining for p53 and Mismatch Repair proteins (MLH1, MSH2, MSH6 and PMS2) was scored on a scale from 0 to 3+ (0, staining completely absent; 1+, immunoreactivity in up to 20% of cells; 2+, immunoreactivity in up to 50% and 3+ up to 51% of cells).

Legend: TC, thyroid cancer; mts, metastasis; TKI, tyrosine kinase inhibitor
