# Supplementary material for: Thyroid cancer harboring PTEN and TP53 mutations: A peculiar molecular and clinical case report
Source: Front Oncol. 2022 Sep 2;12:949098. doi: 10.3389/fonc.2022.949098 (PMC9478947; doi:10.3389/fonc.2022.949098)
Supplement: Supplementary file 5 [file Table_2.docx]

**Supplementary Table 2**: Microsatellite analysis showed that almost all samples were microsatellite stable (MSS). Lymph node metastasis obtained after Sorafenib treatment resulted unstable for two loci (MSI), while the amplification of BAT25 and BAT26 loci failed in the primary tumor sample.

| **Sample Type** | **D17S250** | **D2S123** | **D5S346** | **BAT26** | **BAT25** |
| --- | --- | --- | --- | --- | --- |
| Primary TC | MSS | MSS | MSS | n.d. | n.d. |
| Lymph-node metastasis (2008) | MSS | MSS | MSS | MSS | MSS |
| Lung metastasis (2008) | MSS | MSS | MSS | MSS | MSS |
| Lymph-node metastasis (2009) | MSS | **MSI** | **MSI** | MSS | MSS |
| Lung metastasis (2009) | MSS | MSS | MSS | MSS | MSS |

Legend: TC, thyroid cancer; MSI, microsatellite instability; MSS, microsatellite stable; n.d, not determined.
